# A Multi-Center, Randomised, Double-Blind, Placebo-Controlled Phase III Clinical Trial Evaluating the Impact of BCG Re-Vaccination on the Incidence and Severity of SARS-CoV-2 Infections among Symptomatic Healthcare Professionals during the COVID-19 Pandemic in Poland—First Results

**DOI:** 10.3390/vaccines10020314

**Published:** 2022-02-17

**Authors:** Hanna Czajka, Paweł Zapolnik, Łukasz Krzych, Wojciech Kmiecik, Lidia Stopyra, Anna Nowakowska, Teresa Jackowska, Dorota Darmochwał-Kolarz, Henryk Szymański, Igor Radziewicz-Winnicki, Artur Mazur

**Affiliations:** 1 College of Medical Sciences, University of Rzeszów, 35-315 Rzeszów, Poland; hanna.czajka@onet.pl (H.C.); drmazur@poczta.onet.pl (A.M.); 2 Department of Anaesthesiology and Intensive Therapy, Faculty of Medical Sciences, Medical University of Silesia, 40-055 Katowice, Poland; lkrzych@sum.edu.pl; 3 St. Louis Provincial Specialist Children’s Hospital, 31-503 Kraków, Poland; iplkmiecik@gmail.com; 4 Department of Infectious Diseases and Paediatrics, Stefan Żeromski Specialist Hospital, 31-913 Kraków, Poland; lidiastopyra@gmail.com; 5 Medical Diagnostics Laboratory, Regional Sanitary-Epidemiological Station, College of Medical Sciences, University of Rzeszów, 35-315 Rzeszów, Poland; annanowakowskamik@gmail.com; 6 Blessed Jerzy Popiełuszko Bielany Hospital, 01-809 Warsaw, Poland; tjackowska@cmkp.edu.pl; 7 Department of Gynaecology and Obstetrics, St. Queen Jadwiga Regional Teaching Hospital No. 2, College of Medical Sciences, University of Rzeszów, 35-315 Rzeszów, Poland; dorotak@mp.pl; 8 Saint Hedwig of Silesia Hospital, 55-100 Trzebnica, Poland; henryktomasz@poczta.onet.pl; 9 Transfiguration of the Lord Praga Hospital, 03-401 Warsaw, Poland; irwinnicki@gmail.com

**Keywords:** COVID-19, BCG, vaccines, clinical trial, SARS-CoV-2, health care

## Abstract

Tuberculosis vaccines (Bacillus Calmette-Guérin, BCG) were introduced 100 years ago and are still recommended by the World Health Organization to prevent the disease. Studies have shown that BCG vaccination can stimulate non-specific immune responses and reduce the incidence of certain diseases. At the beginning of the coronavirus disease 2019 (COVID-19) pandemic, it was hypothesised that the incidence of COVID-19 was lower in countries with BCG prevention. In an attempt to verify this thesis, we conducted a multicenter, randomised, double-blind, placebo-controlled study on a group of 695 health care workers aged 25 years and over in Poland. All participants in the study had a tuberculin test, after which those who were negative were randomised (1:1) and received either the BCG- or placebo vaccine. From then on, these people were subjected to three months of observation for the occurrence of COVID-19 symptoms. The statistical analysis did not reveal any significant correlation between the frequency of incidents suspected of COVID-19 and BCG-10 vaccination, the result of the tuberculin test and the number of scars. The only statistically significant feature was the type of medical profession—nurses became infected more often than doctors or other medical workers (*p* = 0.02). The results differ from similar trials in other countries. Perhaps this is due to the lack of an unvaccinated control group. The impact of BCG vaccination on the course of COVID-19 requires further research.

## 1. Introduction

Vaccinations against *Mycobacterium tuberculosis* infections (Bacillus Calmette-Guérin, BCG) were introduced in 1921. The World Health Organisation (WHO, Geneva, Switzerland) still recommend them in the countries characterised by high tuberculosis (TB) incidence rates. Prevention is carried out in European countries, including Poland. Since 1956, the BCG vaccine was administered in Poland several times, and since 2006, we have administered one dose of the vaccine in the first 24 h of life [1,2,3]. The research and observations, mainly in Africa, proved that BCG vaccination is associated with a reduced incidence of infectious diseases and mortality in children with low birth weight. This effect was related to enhancing the immune response and the production of pro-inflammatory cytokines due to immune stimulation after BCG vaccination [4,5,6,7,8,9].

During the COVID-19 pandemic, researchers hypothesised that countries with no widespread tuberculosis prevention have a higher rate of severe disease than countries with long-standing tuberculosis prophylaxis [10]. Clinical trials have been launched in several countries to evaluate the validity of this assumption. Since in Poland BCG vaccinations are carried out universally and compulsorily since the 1950s, it seemed appropriate to assess the impact of these prevention activities on the course of COVID-19 cases in our country. Similarly, as in the other countries that have already started the research, it seems justified to conduct a clinical trial in the same socio-occupational group, especially since the data published by the Chief Sanitary Inspector on 3 April 2020, demonstrate that the number of medical personnel with confirmed coronavirus infection was 461 and 4577 healthcare professionals were quarantined. This group was in contact with afflicted people and became infected more often.

The aim of the study was to assess re-vaccination against tuberculosis with the BCG-10 vaccine (Biomed Lublin S. A., Lublin, Poland) on an impact on SARS-CoV-2 virus infection and the course of COVID-19 disease (incidence, severity) in healthcare workers with a history of BCG vaccination.

## 2. Materials and Methods

### 2.1. Methods

The multicenter, randomised, double-blind placebo-controlled trial was conducted in six centres: Rzeszów, Kraków, Katowice, Warsaw (2 centres), Trzebnica in a group of volunteer health care workers (physicians, nurses, midwives, paramedics, laboratory diagnosticians, electroradiology technicians, physiotherapists, nutritionists, and orderlies). Enrollment occurred from 9 of July 2020 to 29 of December 2020. The Bioethics Committee of the University of Rzeszów approved the study (No. 01/05/2020 of 06/05/2020). Written informed consent was obtained from participants of the study before enrolment. The study was registered at ClinicalTrials.gov (NCT04648800).

The inclusion and exclusion criteria are presented in Table 1.

### 2.2. Intervention

#### 2.2.1. Stage 1

Tuberculin skin tests employing RT 23 were used to evaluate the effects of BCG immunisation. All participants in the study were tested for RT23 using Tuberculin PPD RT 23 SSI. After 72 h, tests were evaluated. Any test causing a local reaction ≥5 mm in diameter was considered positive. On the assessment day, participants with negative test results were randomised (1:1) and received either the BCG-10 vaccine produced by BIOMED-Lublin SA or a placebo. In the first stage of the study, all participants were followed for three-month observation with weekly telephone contact using a standardised checklist to establish their health condition. If symptoms that might indicate infection with the SARS-CoV-2 virus appeared, nasopharynx swabs and blood samples were collected.

Whenever the epidemiologic tuberculosis situation improved, the Polish preventive vaccination program gradually reduced the number of BCG vaccine doses since 1980 from the original 6–7 doses, by 3 or 4 doses in the 1980s, to one dose since 2006. This means that every person over 25 years of age has received more than one dose of the BCG vaccine in Poland.

#### 2.2.2. Stage 2

After introducing universal vaccination against COVID-19, the volunteers participating in the first stage of the study were blood drawn within 1–2 months after the second dose of the Comirnaty vaccine (Pfizer/BioNTech, New York, NY, USA/Mainz, Germany). The study plan is presented in Table 2.

#### 2.2.3. Outcome Measures

The primary outcome was the occurrence of SARS-CoV-2 infection in healthcare workers, confirmed by detecting the genetic material of the virus by PCR (the method described further in the text, Section 2.2). The secondary outcomes included: the number of PCR confirmed SARS-CoV-2 infections in the tuberculin negative, positive, and strong positive group; the number of PCR-confirmed SARS-CoV-2 infections in participants with scars after BCG vaccination; the number of PCR-confirmed SARS-CoV-2 infections in individual health care worker groups.

#### 2.2.4. Allocation and Blinding

The computed randomisation scheme was generated by the e-CRF system. The randomisation lists were stratified according to tuberculin test results—participants with positive results were not randomised and were automatically assigned to group I. The allocation sequence was secured. The study products (vaccine and placebo) were administered intracutaneously in the same volume—0.1 mL. Researchers, participants, outcome assessors and people responsible for the statistical analysis were blinded to the intervention until the completion of the I stage of the study (V5) and data analysis.

### 2.3. Laboratory Tests

Blood for laboratory tests was tested by quantitative enzyme-linked immunosorbent assay (ELISA) to determine the level of IgG antibodies against the S1 antigen of the SARS-CoV-2 virus. Serological examination was performed on the Analyzer I-2P immunological analyzer (EUROIMMUN Medizinische Labordiagnostika AG, Lübeck, Germany), the commercial anti-SARS-CoV-2 QuantiVac ELISA IgG assay was used (EUROIMMUN Medizinische Labordiagnostika AG, Lübeck, Germany). The ELISA reaction wells were coated with the SARS-CoV-2 S protein (spike) S1 domain. In the first stage of identifying IgG antibodies, patients’ sera were diluted in a dedicated buffer in the ratio 1:101. If the extinction of the test serum exceeded the value of 120 RU/mL (relative units/mL, the highest reading value on the calibration curve), further 10-fold dilutions were made, i.e., 1:1010, 1:10,100, etc. (Analyzer I-2P). We adopted the following criteria and interpretation of the performed analyses to evaluate serum samples’ extinction: <8 RU/Ml—negative result; ≥8 to <11 RU/mL—borderline result; ≥11 RU/mL—positive result. In addition to the relative units/mL, we have also given the analysis results in BAU/mL (binding antibody units/mL). They are recognised as the standard of international units (First WHO International Standard for anti-SARS-CoV-2 immunoglobulin. 2021. https://www.who.int/groups/expert-committee-on-biological-standardization (accessed on 7 October 2021)). To evaluate the extinction of serum samples in new units, we have adopted the following criteria: <25.60 BAU/mL—negative result; ≥25.60 to <35.20 BAU/mL—borderline result; ≥35.20 BAU/mL—positive result.

### 2.4. Virological Methods

The central laboratory performed the identification of SARS-CoV-2 virus RNA in nasopharynx swabs from participants suspected of COVID-19. The swabs were collected in centres by researchers or paramedics in participants’ homes. The collected genetic material placed in the stabilising medium was deep-frozen at minus 80 °C/minus 70 °C until delivered to the central laboratory. The transport of the material took place in the conditions of minus 20 °C and did not last longer than 6 h. A commercial kit STARMag 96 × 4 Viral DNA/RNA 200 C Kit (Seegene Inc., Taewon Bldg., 91 Ogeum-ro, Songpa-gu, Seoul, Korea) was used to isolate the coronavirus nucleic acid.

The isolation process was performed automatically using the Microlab NIMBUS IVD (Seegene Inc., Taewon Bldg., 91 Ogeum-ro, Songpa-gu, Seoul, Korea). The automated nucleic acid purification technique was used in the isolation process based on the reversible adsorption of nucleic acids on magnetic beads under appropriate buffer conditions. A complex, one-step commercial kit was used for nucleic acid detection—Allplex™ SARS-CoV-2 Assay (Seegene Inc., Taewon Bldg., 91 Ogeum-ro, Songpa-gu, Seoul, Korea). The detection step was performed automatically on a CFX96 ™ Real-time PCR Detection System (Bio-Rad, Warsaw, Poland) with the appropriate CFX Manager ™ Software-IVD v1.6. The real-time reverse transcription PCR technique was used to identify SARS-CoV-2 RNA in the collected material. The Allplex™ SARS-CoV-2 Assay multiplex assay detected the RdRP/S gene region (RNA dependent RNA polymerase gene)/S gene (spike protein gene), E gene (envelope protein gene) and the N gene (nucleocapsid protein gene, nucleocapsid) using the following fluorophores: Cal Red 610, FAM, Qusar 670 and HEX (for internal control, IC), respectively. The test’s detection limit was determined as 1 GE/μL (the amount of DNA in 1 μL of the purified sample), which guaranteed the detection of all tested SARS-CoV-2 gene regions. The final result of the analysis was given according to the principle: positive result—detection of 3 tested gene regions: RdRp/S, N, E or detection of at least one of the distinct SARS-CoV-2 gene regions: RdRp/S or N; equivocal result—it meant that only the E gene was detected, which did not exclude or confirm the infection caused by SARS-CoV-2; a negative result indicated that the tested regions of the RdRp/S, N, E genes were not detected.

RNA isolates that met the criterion of Ct ≤ 30 for all detected genes in the amplification reading were used for the sequencing of the SARS-CoV-2 virus genome. Sequencing in nanopore technology was performed on the GridIONx5 (Oxford Nanopore Technologies, Oxford, UK). In the first step, the cDNA template was prepared by reverse transcription using a set of reagents NEBNext ARTIC SARS-CoV-2 Companion Kit (NEW ENGLAND BioLabs Inc., Ipswich, MA, USA). In the second step, the obtained cDNA fragment was subjected to the polymerase chain reaction using specific primers complementary to the template DNA. Both stages were performed in a thermocycler Mastercycler nexus (Eppendorf AG, Hamburg, Germany). The PCR products were purified using magnetic beads in the next step, and the DNA concentration was measured on the Qubit 4 Invitrogen (Thermo Fisher Scientific, Waltham, MA, USA) fluorimeter. Samples with DNA concentration ≥ 10 ng/μL were appropriately diluted and barcoded, i.e., the determination of short DNA sequences in the selected, standard genome region with a specific barcode by Oxford Nanopore Technologies. Subsequently, the ligation of the protein adapters and purification with magnetic beads were performed. The amplicons library prepared in this way was placed on the FlowCell by Oxford Nanopore, which started the process of proper sequencing. The results were assessed through a detailed bioinformatics analysis by mapping the readings against a reference sequence using the ARTIC Network pipeline. Identification of the SARS-CoV-2 genetic line of the studied genomes (samples) was facilitated using the PANGOLIN software (https://cov-lineages.org/resources/pangolin.html (accessed on 7 October 2021)). The analysis of the obtained viral genome sequences allowed the detection of the full spectrum of nucleotide changes to the reference sequence, indicating specific mutations of the virus and determining a specific SARS-CoV-2 variant.

### 2.5. Statistical Analysis

Statistical analysis was performed using the procedures available in the licensed MedCalc v17.7. software (MedCalc Software Ltd., Ostend, Belgium). Quantitative variables were presented as an arithmetic mean and standard deviation (normally distributed) or median and interquartile range (skewed variables). The Shapiro–Wilk test was applied to assess the type of distribution. Qualitative variables were presented in the form of absolute value and percentage. Between-group differences for qualitative data were verified using the chi-square test or the Fisher’s exact test. A *p*-value of <0.05 was considered statistically significant.

## 3. Results

**Figure 1:**
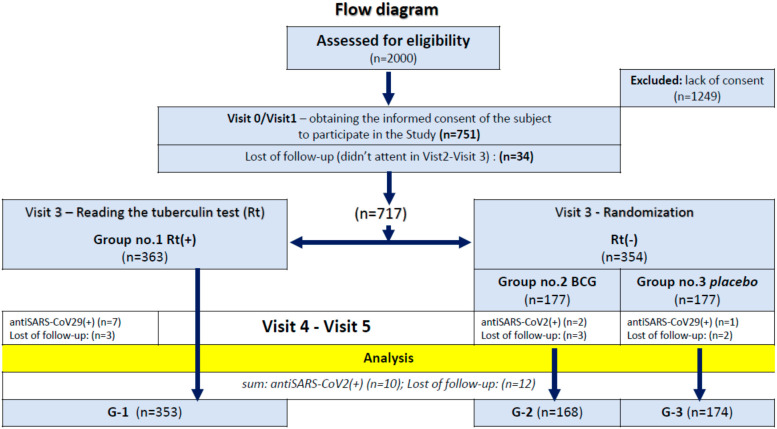
Flow diagram.

During the enrollment period, a total of around 2000 participants were potentially eligible, 717 of whom met all inclusion criteria. Tuberculin test results were positive in 363 (50.6%) participants, 177 of them were randomly assigned to receive the BCG vaccine, and 177 were to receive a placebo (Figure 1). The baseline characteristics of study groups are shown in Table 3, Table 4, Table 5 and Table 6.

SARS-CoV-2 infection events occurred in 161 (23.16%) participants, with asymptomatic seroconversion occurring in 87 people (Table 7). A serious adverse event (SAE) with hospitalisation for COVID-19 occurred in one female participant, 57 years old, who had laboratory-confirmed SARS-CoV-2 infection on 24 October 2020. The patient was hospitalised from 28 October 2020 to 9 November 2020. After the treatment, the patient’s health improved, and she did not require intensive care. The study participant, after a two-week hospitalisation and post-hospital period of inability to work, lasting until 20 November 2020, on 11 December 2020, came to visit no. 5 (V5) in good general condition.

Of the 695 participants, COVID-19 events occurred in 161 participants among the BCG vaccinated people (23.16%) and was absent in 534 (76.84%) of the BCG non-vaccinated group. COVID-19 related events occurred in 79 subjects in group 1 (positive tuberculin test), 38 in group 2 (negative tuberculin test, received BCG-10 vaccine after randomisation) and 44 in group 3 (negative tuberculin test, received placebo after randomisation). The results are presented in the following tables (Table 8, Table 9, Table 10 and Table 11).

There were no statistically significant differences between the treatment groups (*p* = 0.7).

Another analysed element was the influence of a positive RT23 test on the occurrence of a COVID-19 related event. There was no significant difference between positive and negative RT23 participants (*p* = 0.7) (Table 12).

Another question relates to the number of scars after BCG and their impact on the number of suspected COVID-19 cases. As in the previous states, the use of the chi-square test showed no statistically significant differences between the number of scars and the incidence of COVID-19 (*p* = 0.8 and *p* = 0.4 for trend) (Table 13).

In addition, we assessed whether any demographic characteristics (age, BMI, working time with the patient, professional group) affect the number of COVID-19 suspected incidents. Among the features mentioned above, we could determine statistical significance only for the profession performed. The most common incidents were among nurses, less often among doctors and the least frequently among other health care workers (*p* = 0.02). The data are presented in Table 14.

We also assessed whether higher IgG levels are observed among BCG vaccinated individuals when seroconversion occurs between V2 and V4/5. Seroconversion occurred in 150 people, but we did not prove a statistical significance (***p*** = 0.98). IgG concentration at V4/5 does not differ between groups (BCG+/vaccinated/placebo) in seroconverted subjects. The data is provided in Table 15 and Table 16.

An additional parameter assessed in the field of antibodies was whether higher IgG levels are observed among those with a positive RT23 sample when seroconversion occurs between V2 and V4/5. IgG concentration at visit V4/5 in seroconverted subjects did not differ between positive and negative RT23 result individuals (*p* = 0.89)—Table 16 and Table 17.

Identification of the SARS-CoV-2 genetic line of the examined genomes (24) was performed using PANGOLIN software (https://cov-lineages.org/resources/pangolin.html (accessed on 10 January 2022)). Among the analysed sequences (tests were conducted from materials collected between 28 October 2020 and 7 December 2020), 10 belonged to the genetic line B.1.258 widely distributed in the world since March 2020; 6 to the line B.1.1.170 occurring in the world since July 2020, mainly in Europe, the rest found B.1.1.277 (2), B.1.1.153 (2), B.1.177 (1), B.1.1 (1), B.1.1.1 (1), B.1.1.8 (1) were also among the lines found in Europe; none of the SARS-CoV-2 genetic lines reported qualified for variants of special epidemic significance (Variants of Concern, Variants of Interest, Variants Under Monitoring, Variants of Note).

## 4. Discussion

The BCG vaccine uses a live-attenuated strain of *Mycobacterium bovis* and is the only licenced vaccine against tuberculosis, which is routinely administered to neonates at birth or shortly after in the regions with endemic tuberculosis. BCG vaccination provides continuous protection against disseminated forms of tuberculosis during childhood, e.g., meningeal tuberculosis and military tuberculosis. However, its protective efficacy against pulmonary tuberculosis in adults is not entirely satisfying.

Many countries carry out BCG prevention policies; in some of them, however, BCG vaccinations have been abandoned due to improved epidemiological situations (Spain 1981, Denmark 1986). Moreover, some countries never conducted this kind of prevention (Canada, USA, Belgium, Italy, The Netherlands) [2].

Although preventive BCG vaccination has a 100-year history, its importance for population immunisation and all the mechanisms it stimulates are not fully elucidated.

During the COVID-19 pandemic, it was hypothesised that countries without widespread tuberculosis prevention policies have a higher percentage of the severe disease course (Italy, France, Spain, The Netherlands) than countries with long-term widespread prevention (Japan 1947, Denmark, Korea). In the countries where widespread tuberculosis prevention was recently introduced (Iran 1984), there are no positive effects of BCG vaccination, as prevention covers individuals up to the age of 36 years. On 24 March 2020, US researchers published an analysis demonstrating that the countries without tuberculosis vaccination programs (Italy, The Netherlands, USA) have higher incidence rates and death rates due to SARS-CoV-2 infections than the countries with widespread and long-term BCG vaccination [10].

In 55 countries with widespread BCG vaccination programs, the mortality rate per 1 million inhabitants associated with COVID-19 is 0.78 ± 0.40. Furthermore, in five countries where BCG vaccination was never widespread, the mortality rates are 16.39 ± 7.33. In Iran, tuberculosis vaccination started in 1984, and the COVID-19 mortality rate is 19.7 per million; in Japan, vaccinations began in 1947, and now the COVID-19 mortality rate is 100 times lower (0.28/1,000,000). Furthermore, in Brazil, BCG vaccination programs started in 1920, and the mortality rate in question is 0.0573/1,000,000. There is also a correlation between the period of widespread vaccinations against tuberculosis and mortality due to COVID-19. For instance, in Spain, BCG vaccinations were used for 16 years (1965–1981), and the mortality rate observed is 25.5/1,000,000; in Denmark, vaccinations were carried out for 40 years (1946–1986), resulting in 10 times fewer deaths/1,000,000 inhabitants [10].

Numerous authors have recently discussed the impact of BCG vaccination on the severity of SARS-CoV-2 infections. Some support Harvard University researchers, while others do not attribute this effect to tuberculosis vaccinations. However, the vast majority of researchers are inclined toward the need for further and broader research to confirm or deny this theory [11,12,13,14,15,16].

Therefore, recently, 22 clinical trials of health care workers were registered (as of 20 January 2021) to determine the importance of BCG vaccinations in protecting against SARS-CoV-2 infections and the possible effects of such vaccinations on the alleviation of COVID-19 [17].

The results of our study presented in this article suggest no statistical significance between the group with the positive and negative tuberculin test results. The incidence of COVID-19 was also similar after randomisation in the placebo and BCG vaccinated groups. The tuberculin reaction size and the size of the scars after vaccination also did not give a statistically significant result.

Currently (2021), available publications describe the results of the first association of BCG vaccination and COVID-19 cases. Weng et al. [18] (Providence, RI, USA) analysed a cohort of 120 adult COVID-19 patients at the beginning of the pandemic in March and April 2020. Eighty-two people (68.3%) were vaccinated with BCG, and, according to the authors, this group had a lower risk of hospitalisation during the disease compared to the unvaccinated. Statistical analysis showed significance at *p* = 0.019. Compared to our study, this work was not a clinical trial but an observation. In this case, the research group also included patients who were never vaccinated with BCG. This fundamental difference may have contributed to the above result as BCG vaccination stimulates the “trained immunity” and stimulates the production of pro-inflammatory cytokines. The never-vaccinated participants may not have obtained the positive effects of BCG vaccine stimulation of the non-specific response. An additional difference between the study mentioned above and ours is that the participants are patients of the federal health centre, not medical workers; they also come from a different population (Latino/Hispanic), which could have influenced the differences in the obtained results.

Rivas et al. [19] analysed over 6000 health care workers in terms of vaccination against tuberculosis. In total, 29.6% of them were vaccinated, while 68.9% did not receive the BCG vaccine. Reporting of COVID-19 disease symptoms and seroprevalence based on the assessment of anti-SARS-CoV-2 IgG antibody titer was significantly lower in the group of health care workers vaccinated with BCG compared to the unvaccinated group. The authors also analysed the study participants concerning vaccination against *Neisseria meningitidis*, *Streptococcus pneumoniae* and influenza but did not obtain similar results [19]. The study group had a similar mean age (43 years) as in our case. Still, it differed essentially in the presence of never vaccinated people and a different population structure (Asians, African Americans, Caucasians, native Hawaiians). It was also not a randomised clinical trial but a retrospective observational study; therefore, the presented results do not match those obtained by us.

Tsilika et al. [20] conducted a randomised, double-blind clinical trial on 516 elderly Greek citizens (median age 68), with the final administration of 301 BCG or placebo and a 6 month follow-up for the occurrence of COVID-19. BCG re-vaccination led to a 68% reduction in the risk of COVID-19 with clinical and microbiological confirmation of the diagnosis (OR 0.32, 95% CI 0.13–0.79). There were six episodes of severe COVID-19 during the study, requiring hospitalisation, of which five patients received a placebo and one person received BCG. The study’s authors suggest that vaccinating elderly patients with the BCG vaccine may be a safe and effective prevention method against COVID-19 [20]. The differences in the results presented by Tsilika et al. concerning our study can be found in the more extended follow-up period and the older age of the target population. They were also not health care workers. An additional limitation of the above study was the lack of microbiological confirmation of SARS-CoV-2 infection, which could have influenced the actual number of infections. The study participants were born when newborns were vaccinated with BCG at birth in Greece, a situation analogous to our work. A different target group, follow-up time and a smaller number of participants are probably the main reasons for the differences between the work mentioned above and ours.

Another worth work is the study from the United Arab Emirates by Amirlak et al. [21]. In March 2020, 280 employees of The Emirates International Hospital were offered BCG (Serum Institute of India PVT. LTD. Hadaspar India) vaccinations to stimulate a non-specific reaction and improve the immune system response. All 280 people had previously been vaccinated against tuberculosis, of which 71 took an additional dose of BCG and 209 did not receive it. During the 3 month follow-up (until June 2020), the authors recorded 18 cases of SARS-CoV-2 infection among hospital staff, including 13 with symptomatic and 5 with the asymptomatic course. All of the cases were in the group that did not receive an additional dose of BCG vaccine. Seventy-one study participants who received vaccinations did not experience local or systemic adverse events. Statistical significance was determined at the level of *p* = 0.004 [21]. In the above-cited study, participants were healthcare workers of a similar age to our sample (21–80 years of age), and all had previously been vaccinated with BCG. However, the results of the statistical analysis differ from our work. Perhaps this is due to the more diverse structure of the population studied by Amirlak et al. (Arab, Indian, European, African, East Asian origin) and the group’s smaller size (280 people).

On the other hand, a review by Labetoulle et al. [22] mentions a study evaluating the incidence of COVID-19 in BCG vaccinated and unvaccinated adult Israelis aged 35–41 years [23]. The authors assessed the results of PCR tests for the presence of SARS-CoV-2 in 3064 people born before and 2869 born after 1982 (end of the general tuberculosis vaccination program in Israel). In this case, the incidence of SARS-CoV-2 infection did not differ between the two groups. Although the study did not include medical workers, the results are similar to our results. Perhaps it is influenced by the relatively similar age structure of the population (mean 43 years), but the above study is not a controlled clinical trial. Therefore, it is difficult to say unequivocally what results the authors would obtain after vaccinating part of the already vaccinated patients.

The only statistically significant feature among those presented in work was the type of medical profession—nurses became infected more often than doctors or other health care workers. Other studies around the world confirm these observations. Gómez-Ochoa et al. [24] prepared a meta-analysis on the incidence of COVID-19 among health care workers. The authors of 4107 reports finally analysed 97 papers on this topic. The group of nurses was characterised by the highest frequency of positive results for the presence of the SARS-CoV-2 coronavirus (48%). Physicians (25%) and other medical workers (23%) were infected less frequently. Our results and other observations are probably due to the longer time nurses spend directly with the patient, such as administering medications, general care or in an emergency situation where the nurse is usually the first to arrive.

The most significant limitation of the study was the lack of a never-vaccinated control population. As mentioned above, BCG vaccination has been obligatory in Poland since 1955; therefore, all surveyed healthcare professionals had previously received at least two doses of the BCG vaccine. Perhaps the study’s control with a never-vaccinated group could lead to a different result and confirm some observations that the incidence of COVID-19 is lower in populations with BCG prophylaxis. An additional limitation could also be the smaller number of participants included in the study—ultimately 717, with the planned 1000. It is worth noting that the stimulation of non-specific effects after the BCG vaccine is associated, among others, with epigenetic modifications in macrophages, which lead to a change in their phenotype to a more pro-inflammatory [7,9,25,26]. The profile of gene expression and epigenetic modifications such as acetylation/methylation of histones may differ between populations, which could have influenced our study results compared to trials conducted in other countries.

Due to the lack of complete knowledge about all routes of influence of the BCG vaccine on the stimulation of the immune system, epidemiological observations and the results of the first cohort and clinical studies, it seems appropriate to explore further the knowledge about the impact of BCG prophylaxis on the incidence and severity of COVID-19 disease in various populations. Additionally, international clinical trials analysing different populations can bring new light to the topic of factors determining the incidence of COVID-19, including BCG vaccination.

## Figures and Tables

**Table 1:** The study inclusion and exclusion criteria.

Inclusion Criteria	Exclusion Criteria
A health care professional (physician, nurse, midwife, paramedic, electroradiology technician, laboratory diagnostician, physiotherapist, nutritionist, orderly) aged > 25 years	Hypersensitivity to any component of BCG-10
No confirmed SARS-CoV-2 infection	Hypersensitivity to previously administered tuberculin (local skin lesions, necrosis of the skin, blisters, other severe skin reactions at the injection site)
Informed consent to participate in the trial and consent to personal data processing	HIV infection (confirmed or suspected infections, even if they are asymptomatic)
Declared availability for telephone contacts throughout the study period	Primary or secondary immunodeficiencies (including interferon-gamma deficiency or DiGeorge syndrome)
Good health condition	Radiotherapy (less than 24 months before the date of inclusion in the trial)
Earlier vaccination against tuberculosis	Treatment with corticosteroids, ongoing immunosuppressive therapy (including those treated with monoclonal antibodies to TNF-α, such as infliximab)—less than 24 months before the date of inclusion in the trial
Receive two doses of the COVID-19 vaccine as part of the National Immunization Program after December 27, 2020	Neoplastic diseases (e.g., leukaemia, malignant granuloma, lymphoma or some other cancer of the reticuloendothelial system)—less than 24 months before the date of inclusion in the study
	After stem cell transplantation and organ transplantation
	In the exacerbation stage of chronic diseases (including severe malnutrition)
	Pregnancy
	History of tuberculosis
	Keloid at the vaccination site after previous BCG vaccination

**Table 2:** The study plan. Patients who did not complete V5 had an antibody level assay performed from V4. Some patients had combined V4 and V5 visits due to the population vaccinations against COVID-19 introduced and the need to accelerate visits.

Stage of the Study	Stage 1							Stage 2	
Visit/Contact	Visit 1 (V1)	Visit 2 (V2)	Visit 3 (V3)	Weekly Phone Call	Visit 4 (V4)	Weekly Phone Call	Visit 5 (V5)	Visit 6 (V6)	Visit 7 (V7)
Signing the informed consent	X							X	
Taking medical history	X							X	X
Setting up tuberculin test (RT23)		X							
Blood sampling		2 × 5 mL			5 mL		2 × 5 mL	2 × 5 mL	2 × 5 mL
Assessment of the tuberculin test			X						
Randomisation			X						
Telephone call				X		X			
Unblinding of the study							X		

**Table 3:** Baseline characteristics with profession structure.

Groups	695		Profession (All)	Number of Participants
**Gender**			Physician	**376**
Women	541	77.8%	Midwife	**29**
Men	154	22.2%	Laboratory diagnostician	**16**
Age (mean + standard deviation)	43.8	11.8	Nurse	**151**
**Profession**			Paramedic	**19**
			Nutritionist	**11**
Physicians	376	54.1%	Physiotherapist	**36**
Nurses/Midwifes/Paramedics	199	28.6%	Electroradiology technician	**10**
Other medical profession	120	17.3%	Orderly	**34**
Average time with the patient (h)	31.5	16.1	Clinical psychologist	**13**
Weekly working time (h)	46.1	14.1	**Total**	**695**

**Table 4:** Characteristics of group 1 with profession structure.

Group 1	353		Profession (Group 1)	Number of Participants
**Gender**			Physician	**194**
Women	264	74.8%	Midwife	**17**
Men	89	25.2%	Laboratory diagnostician	**6**
Age (mean + standard deviation)	42.2	11.3	Nurse	**78**
**Profession**			Paramedic	**12**
			Nutritionist	**6**
Physicians	194	55.0%	Physiotherapist	**18**
Nurses/Midwifes/Paramedics	107	30.3%	Electroradiology technician	**6**
Other medical profession	52	14.7%	Orderly	**12**
Average time with the patient (h)	32.5	15.7	Clinical psychologist	**4**
Weekly working time (h)	46.9	13.6	**Total**	**353**

**Table 5:** Characteristics of group 2 with profession structure.

Group 2	168		Profession (Group 2)	Number of Participants
**Gender**			Physician	**89**
Women	127	75.6%	Midwife	**6**
Men	41	24.4%	Laboratory diagnostician	**6**
Age (mean + standard deviation)	46.3	12.1	Nurse	**34**
**Profession**			Paramedic	**2**
			Nutritionist	**2**
Physicians	89	53.0%	Physiotherapist	**9**
Nurses/Midwifes/Paramedics	42	25.0%	Electroradiology technician	**2**
Other medical profession	37	22.0%	Orderly	**13**
Average time with the patient (h)	30.5	17.1	Clinical psychologist	**5**
Weekly working time (h)	45.6	14.0	**Total**	**168**

**Table 6:** Characteristics of group 3 with profession structure.

Group 3	174		Profession (Group 3)	Number of Participants
**Gender**			Physician	**93**
Women	150	86.2%	Midwife	**6**
Men	24	13.8%	Laboratory diagnostician	**4**
Age (mean + standard deviation)	44.7	11.8	Nurse	**39**
**Profession**			Paramedic	**5**
			Nutritionist	**3**
Physicians	93	53.5%	Physiotherapist	**9**
Nurses/Midwifes/Paramedics	50	28.8%	Electroradiology technician	**2**
Other medical profession	31	17.8%	Orderly	**9**
Average time with the patient (h)	30.4	15.9	Clinical psychologist	**4**
Weekly working time (h)	45.1	15.1	**Total**	**174**

**Table 7:** The analysis of seroconversion in SARS-CoV-2 event participants.

Disease Incidents	Seroconversion Analysis				Symptomatic Seroconversion
	Number of PCR Tests	Positive (+)	Equivocal Result (+/−0+)	Negative (−)	
79	64	38	1	25	39
38	27	21	1	5	22
44	32	18	0	14	26
**Total: 161**	**123**	**77**	**2**	**44**	**87**

**Table 8:** Comparison of the number of COVID-19 related events by treatment group.

Did a COVID-19 Event Occur during the Observation?	Group 1	Group 2	Group 3	Total:
** YES **	79	38	44	** 161 ** (23.16%)
** Total **	** 353 ** (50.80%)	** 168 ** (24.17%)	** 174 ** (25.03%)	** 695 **

Group 1—a positive result of the tuberculin test. Group 2—received BCG. Group 3—received placebo.

**Table 9:** Percentage of COVID-19 disease incidents by the medical profession.

Profession	N	%	Disease Incidents	% of Group with Incidents	V4/5 Negative	V4/5 Positive	% of the Positive Patients
**Physicians**	376	54.1%	82	21.8%	309	67	17.8%
**Nurses/Midwifes/Paramedics**	199	28.6%	58	29.2%	136	63	31.7%
**Other medical profession**	120	17.3%	21	17.5%	100	20	16.7%
**Total amount**	**695**	**100.0%**	**161**	**23.2%**	**545**	**150**	**21.6%**

**Table 10:** Division of the study participants into three treatment groups.

Patients Group	Amount	%	V2 Negative	V2 Positive
**Group 1 (RT23+)**	360	51.1%	353	7
**Group 2 (BCG)**	170	24.1%	168	2
**Group 3 (Placebo)**	175	24.8%	174	1
**Total amount**	705	100.0%	695	10

**Table 11:** Percentage of positive seroconversion among the treatment groups.

Seropositive Patients Were Excluded at Baseline (V2)							
Patients Group	Amount	V4/5 Negative	V4/5 Positive	% of the Positive Patients	Asymptomatic Seroconversion	Symptomatic Seroconversion	Disease Incidents
**Group 1 (RT23+)**	353	282	71	20.1%	32	39	79
**Group 2 (BCG)**	168	128	40	23.8%	18	22	38
**Group 3 (Placebo)**	174	135	39	22.4%	13	26	44
**Total amount**	695	545	150	21.6%	63	87	161

**Table 12:** The number of COVID-19 incidents depending on the result of the RT23 test.

Did a COVID-19 Event Occur during the Observation?	RT23 Test Result			Total
	Negative	Positive	Strongly Positive	
** YES **	82	64	15	** 161 ** (23.16%)
** TOTAL **	** 342 ** (49.21%)	** 297 ** (42.73%)	** 56 ** (8.06%)	** 695 **

**Table 13:** The number of COVID-19 incidents depending on the number of scars after BCG vaccination.

Did a COVID-19 Event Occur during the Observation?	Number of Scars					Total
	0	1	2	3	4	
YES	5	78	59	16	3	** 161 ** (30.15%)
** TOTAL **	** 18 ** (3.37%)	** 233 ** (43.63%)	** 209 ** (39.14%)	** 62 ** (11.61%)	** 12 ** (2.25%)	** 534 **

**Table 14:** The frequency of COVID-19 incidents by profession.

Did a COVID-19 Event Occur during the Observation?	Profession			Total:
	Physician	Nurse, Midwife, Paramedic	Other	
** YES **	** 82 **	** 58 **	** 21 **	** 161 ** (23.17%)
** TOTAL **	** 376 ** (54.10%)	** 199 ** (28.63%)	** 120 ** (17.27%)	** 695 **

**Table 15:** The seroconversion between visits V2 and V4/5.

Seroconversion	Interpretation V2		Total
Interpretation V4/V5	Negative	Positive	
** Negative **	545 (99.8%) RT 78.4% CT 77.3% GT	10.2% RT 10.0% CT 0.1% GT	546 (77.4%)
** Positive **	**150** **(94.3%) RT** **21.6% CT** **21.3% GT**	95.7% RT 90.0% CT 1.3% GT	159 (22.6%)
** Total **	695 (98.6%)	10 (1.4%)	705

**Table 16:** The comparison of antibody levels between the three groups in the study.

Factor	*n*	Minimum [BAU/mL]	25th Percentile [BAU/mL]	Median [BAU/mL]	75th Percentile [BAU/mL]	Maximum [BAU/mL]
**Group 1**	71	36.8900	84,465	129,950	275,405	10,905,710
**Group 2**	40	36.5400	60,620	123,825	385,565	1,209,760
**Group 3**	39	36.4800	80,762	142,300	310,225	5,245,030

**Table 17:** The comparison of antibody levels between participants with positive and negative RT23 test results.

Factor	*n*	Minimum [BAU/mL]	25th Percentile [BAU/mL]	Median [BAU/mL]	75th Percentile [BAU/mL]	Maximum [BAU/mL]
**RT23 negative**	79	36.4800	65,363	141,400	353,727	5,245,030
**RT23 positive**	71	36.8900	84,465	129,950	275,405	10,905,710

## Data Availability

EudraCT No. 2020-002111-22.

## Abbreviations

BCG	Bacillus Calmette–Guérin
cDNA	complementary DNA
COVID-19	Coronavirus disease 2019
DNA	Deoxyribonucleic acid
HIV	Human immunodeficiency virus
IFN-γ	Interferon gamma
IgG	Immunoglobulin G
IL-1β	Interleukin 1 beta
IL-5	Interleukin 5
IL-6	Interleukin 6
IL-10	Interleukin 10
IL-13	Interleukin 13
PCR	Polymerase chain reaction
RNA	Ribonucleic acid
SAE	Serious adverse event
SARS-CoV-2	Severe acute respiratory syndrome coronavirus 2
TB	Tuberculosis
TNF-α	Tumour necrosis factor-alpha
WHO	World Health Organisation

## References

[1] World Health Organization (2011). Recommendations to Assure the Quality, Safety and Efficacy of BCG Vaccines.

[2] Zwerling A., Behr M.A., Verma A., Brewer T.F., Menzies D., Pai M. (2011). The BCG World Atlas: A database of global BCG vaccination policies and practices. PLoS Med..

[3] Magdzik W., Naruszewicz-Lesiuk D., Zieliński A. (2007). Wakcynologia.

[4] Garly M.L., Martins C.L., Balé C., Baldé M.A., Hedegaard K.L., Gustafson P., Lisse I.M., Whittle H.C., Aaby P. (2003). BCG scar and positive tuberculin reaction associated with reduced child mortality in West Africa. A non-specific beneficial effect of BCG?. Vaccine.

[5] Stensballe L.G., Sørup S., Aaby P., Benn C.S., Greisen G., Jeppesen D.L., Birk N.M., Kjærgaard J., Nissen T.N., Pihl G.T. (2017). BCG vaccination at birth and early childhood hospitalisation: A randomised clinical multicentre trial. Arch. Dis. Child..

[6] Aaby P., Roth A., Ravn H., Napirna B.M., Rodrigues A., Lisse I.M., Stensballe L., Diness B.R., Lausch K.R., Lund N. (2011). Randomized trial of BCG vaccination at birth to low-birth-weight children: Beneficial nonspecific effects in the neonatal period?. J. Infect. Dis..

[7] Netea M.G., Joosten L.A., Latz E., Mills K.H., Natoli G., Stunnenberg H.G., O’Neill L.A., Xavier R.J. (2016). Trained immunity: A program of innate immune memory in health and disease. Science.

[8] Badurdeen S., Marshall A., Daish H., Hatherill M., Berkley J.A. (2019). Safety and Immunogenicity of Early Bacillus Calmette-Guérin Vaccination in Infants Who Are Preterm and/or Have Low Birth Weights: A Systematic Review and Meta-analysis. JAMA Pediatr..

[9] Leentjens J., Kox M., Stokman R., Gerretsen J., Diavatopoulos D.A., van Crevel R., Rimmelzwaan G.F., Pickkers P., Netea M.G. (2015). BCG Vaccination Enhances the Immunogenicity of Subsequent Influenza Vaccination in Healthy Volunteers: A Randomized, Placebo-Controlled Pilot Study. J. Infect. Dis..

[10] Hensel J., McAndrews K.M., McGrail D.J., Dowlatshahi D.P., LeBleu V.S., Kalluri R. (2020). Exercising caution in correlating COVID-19 incidence and mortality rates with BCG vaccination policies due to variable rates of SARS CoV-2 testing. medRxiv.

[11] SSRN The Correlation between BCG Immunization Coverage and the Severity of COVID-19.

[12] ResearchGate Association between BCG Policy and COVID19 Infection Rates Is Significantly Confounded by Age and Is Unlikely to Alter Infection or Mortality Rates. https://www.researchgate.net/publication/340463940.

[13] Shet A., Ray D., Malavige N., Santosham M., Bar-Zeev N. (2020). Differential COVID-19-attributable mortality and BCG vaccine use in countries. medRxiv.

[14] Devi D., Saniya Gupta S. (2020). Connecting BCG Vaccination and COVID-19: Additional Data. medRxiv.

[15] Kurthkoti K., Das G. (2020). Mechanism of heterologous resistance of BCG to COVID-19. https://osf.io/f32pz/.

[16] Berg M.K., Yu Q., Salvador C.E., Melani I., Kitayama S. (2020). Mandated Bacillus Calmette-Guérin (BCG) vaccination predicts flattened curves for the spread of COVID-19. Sci. Adv..

[17] ClinicalTrials.gov. https://clinicaltrials.gov/ct2/results?pg=1&load=cart&id=NCT04347876+OR+NCT04350931+OR+NCT04327206+OR+NCT04328441+OR+NCT04348370.

[18] Weng C.H., Saal A., Butt W.W., Bica N., Fisher J.Q., Tao J., Chan P.A. (2020). Bacillus Calmette-Guérin vaccination and clinical characteristics and outcomes of COVID-19 in Rhode Island, United States: A cohort study. Epidemiol. Infect..

[19] Rivas M.N., Ebinger J.E., Wu M., Sun N., Braun J., Sobhani K., Van Eyk J.E., Cheng S., Arditi M. (2021). BCG vaccination history associates with decreased SARS-CoV-2 seroprevalence across a diverse cohort of health care workers. J. Clin. Investig..

[20] Tsilika M., Taks E., Dolianitis K., Kotsaki A., Leventogiannis K., Damoulari C., Kostoula M., Paneta M., Adamis G., Papanikolaou I.C. (2021). Activate-2: A Double-Blind Randomized Trial Of Bcg Vaccination Against Covid19 In Individuals At Risk. medRxiv.

[21] Amirlak L., Haddad R., Hardy J.D., Khaled N.S., Chung M.H., Amirlak B. (2021). Effectiveness of booster BCG vaccination in preventing COVID-19 infection. Hum. Vaccines Immunother..

[22] Labetoulle R., Detoc M., Gagnaire J., Berthelot P., Pelissier C., Fontana L., Botelho-Nevers E., Gagneux-Brunon A. (2020). COVID-19 in health-care workers: Lessons from SARS and MERS epidemics and perspectives for chemoprophylaxis and vaccines. Expert Rev. Vaccines.

[23] Hamiel U., Kozer E., Youngster I. (2020). SARS-CoV-2 Rates in BCG-Vaccinated and Unvaccinated Young Adults. JAMA.

[24] Gómez-Ochoa S.A., Franco O.H., Rojas L.Z., Raguindin P.F., Roa-Díaz Z.M., Wyssmann B.M., Guevara S., Echeverría L.E., Glisic M., Muka T. (2021). COVID-19 in Health-Care Workers: A Living Systematic Review and Meta-Analysis of Prevalence, Risk Factors, Clinical Characteristics, and Outcomes. Am. J. Epidemiol..

[25] Kleinnijenhuis J., Quintin J., Preijers F., Joosten L.A., Ifrim D.C., Saeed S., Jacobs C., van Loenhout J., de Jong D., Stunnenberg H.G. (2012). Bacille Calmette-Guerin induces NOD2-dependent nonspecific protection from reinfection via epigenetic reprogramming of monocytes. Proc. Natl. Acad. Sci. USA.

[26] Arts R., Moorlag S., Novakovic B., Li Y., Wang S.Y., Oosting M., Kumar V., Xavier R.J., Wijmenga C., Joosten L. (2018). BCG Vaccination Protects against Experimental Viral Infection in Humans through the Induction of Cytokines Associated with Trained Immunity. Cell Host Microbe.

